# First person – Jeanne Rakotopare

**DOI:** 10.1242/dmm.050488

**Published:** 2023-10-18

## Abstract

First Person is a series of interviews with the first authors of a selection of papers published in Disease Models & Mechanisms, helping researchers promote themselves alongside their papers. Jeanne Rakotopare is first author on ‘ A systematic approach identifies p53-DREAM pathway target genes associated with blood or brain abnormalities’, published in DMM. Jeanne is a PhD student in the lab of Franck Toledo at Institut Curie, Paris, France, investigating the impact of p53 deregulation in bone marrow syndromes and cancer.


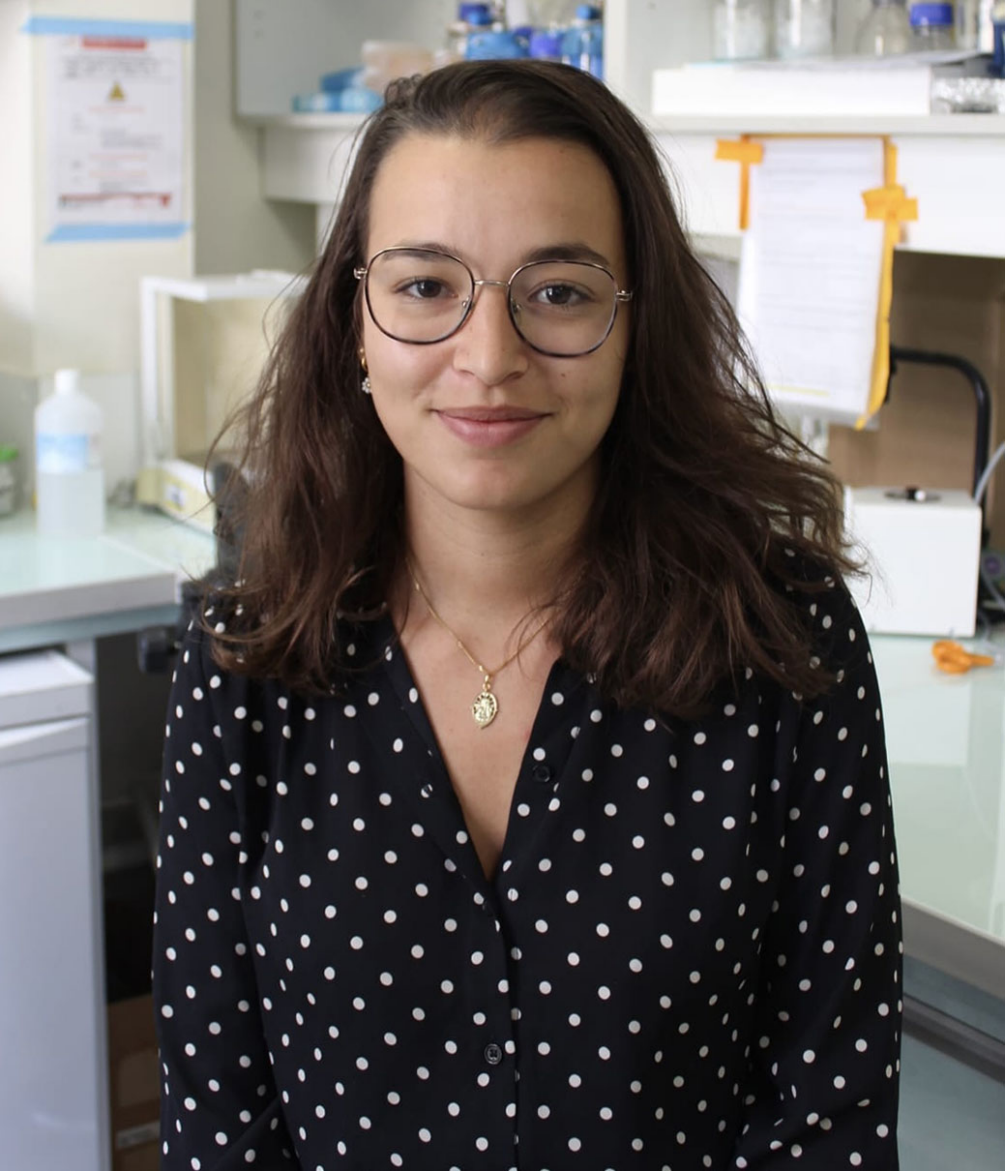


**Jeanne Rakotopare**

**How would you explain the main findings of your paper to non-scientific family and friends?**

Mutations that inactivate the p53 protein are found in about half of human cancers. p53 is well known to suppress tumor growth and to activate the transcription of genes involved in many cellular processes, such as proliferation arrest or cell death. In contrast, our lab showed that mice with a hyperactive mutant of p53 have anemia and brain hypoplasia, and p53's capacity to repress genes indirectly via the DREAM repressor complex appeared to be important for these pathologies. In our study, we designed a systematic approach, combining computational and experimental methods, for the identification of p53-DREAM pathway target genes for which repression might contribute to blood or brain abnormalities. Our method provides a resource of 151 potential DREAM targets, of which 106 are mutated in patients that have a blood or brain genetic disorder. Our findings indicate a crucial role for the p53-DREAM pathway in regulating hematopoiesis and brain development. They not only improve our understanding of the mechanisms implicated in severe and complex pediatric diseases known as ‘inherited bone marrow failure syndromes’, but also provide clues about glioblastoma, an aggressive type of brain cancer.

[Our findings] not only improve our understanding of the mechanisms implicated in severe and complex pediatric diseases […], but also provide clues about glioblastoma, an aggressive type of brain cancer.

**What are the potential implications of these results for your field of research?**

The importance of p53-dependent transcriptional repression emerged recently. Our systematic approach provides a resource of predicted DREAM-binding sites for genes associated with blood and brain abnormalities. Many of the genes in our list of predicted DREAM targets were not known to be a DREAM target. This study underlines the role of the p53-DREAM pathway in bone marrow failure syndromes, neurodevelopmental disorders and cancer. Our results provide an explanation for the variety of clinical symptoms that might result from its deregulation. This could be useful for researchers and doctors interested in blood and neurological diseases. Furthermore, our approach could be applied to find genes associated with other pathologies.

**What are the main advantages and drawbacks of the experimental system you have used as it relates to the disease you are investigating?**

To find a large number of p53-DREAM targets involved in blood or brain abnormalities, we carried out our study with computational methods. One of the advantages of this approach is that we were able to find and use data specific to blood or brain cells. We designed a systematic approach with different steps and criteria, including the analysis of transcriptomic data associated with bone marrow cell differentiation and p53 activation using Gene Ontology analysis, chromatin immunoprecipitation-sequencing (ChIP-seq) data to find promoters bound by the DREAM complex, and also RNA-sequencing (RNAseq) data from irradiated hematopoietic stem cells or splenic cells and from Zika virus-infected neural progenitors. All these different datasets increased the biological relevance of our analysis.

To identify the DREAM-binding sites (DBSs) in the promoter of candidate target genes, we used positional frequency matrices based on functionally demonstrated DBSs. After the validation of several new DBSs, our matrix was improved by including these additional sequences to create a new consensus sequence. We are aware that a main limitation of this method is that we did not validate all the DBSs identified experimentally, but we did find some previously demonstrated genes like *Fancd2*, and we tested and validated 21 DBSs by luciferase assays, which shows the robustness of our approach.

**Figure:**
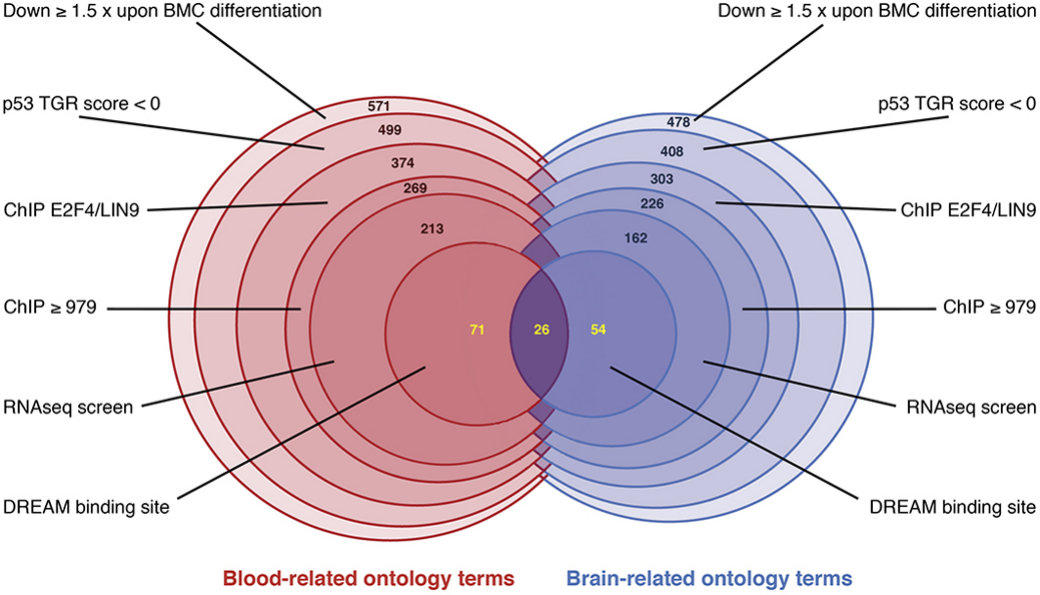
**Venn-like diagram of our systematic approach.** We used a multi-step approach to identify p53-DREAM pathway target genes implicated in blood or brain genetic disorders. Gene Ontology analysis was first used to search for clinically relevant genes downregulated at least 1.5 times upon murine bone marrow cell differentiation. Among these genes, we selected those reported to be downregulated by p53 according to the TargetGeneRegulation database, and the promoters of which were bound by two subunits of the DREAM complex (E2F4 and LIN9). RNAseq data, notably from bone marrow cells and neural progenitor cells, helped us to identify the best candidates, and we identified DREAM-binding sites for 151 of these genes. Numbers in black indicate genes related to either blood- or brain-related ontology terms, which were analyzed separately. In the last step, numbers in yellow indicate genes related to blood-related terms only, brain-related terms only, or both blood- and brain-related terms.

**What has surprised you the most while conducting your research?**

I was impressed by the quantity and quality of public datasets available that could be resourceful for further research and metanalyses.

Concerning my results, I was surprised to see that some genes in our list of candidate genes were not repressed in the dataset from blood cells but repressed in the datasets from brain cells. This suggests a complex, tissue-specific gene regulation by the p53-DREAM pathway, which deserves further investigation.

**What do you think is the most significant challenge impacting your research at this time and how will this be addressed over the next 10 years?**

Research in rare diseases such as inherited bone marrow failure syndromes (dyskeratosis congenita, Fanconi anemia or Diamond–Blackfan anemia) is difficult to comprehend because there are insufficient patient data. That is why I think increased and encouraged collaborations between scientists and doctors will be important in the coming years.

Furthermore, *TP53* is one of the most studied genes, involved in many different pathological processes. However, the importance of the p53-DREAM pathway emerged only recently, and how this pathway operates is not fully understood. For example, the DREAM repressor complex was reported to bind promoters with a single or a bipartite binding motif, which adds further complexity. In the future, I hope we will gain a better understanding of the role of the p53-DREAM pathway in cellular processes and diseases.

[…] there is still room for improvement regarding the consideration of ‘negative’ results in science. Indeed, they allow researchers to discard hypotheses.

**What changes do you think could improve the professional lives of scientists?**

If we want to keep people motivated and ambitious in research, it is important to reform and improve job opportunities and job security, especially for young scientists.

In addition, there is still room for improvement regarding the consideration of ‘negative’ results in science. Indeed, they allow researchers to discard hypotheses. Furthermore, if researchers share them, it could avoid repetition of failed experiments and might encourage collaborations between teams with similar problems.

**What's next for you?**

I plan to continue studying the role of the p53-DREAM pathway with a new mouse model, to complete another manuscript by the end of my PhD. After my thesis defense in September 2024, I would like to continue in research as a postdoc.
